# Technical Brief: A comparison of two methods of euthanasia on retinal dopamine levels

**Published:** 2013-05-29

**Authors:** Christopher K. Hwang, P. Michael Iuvone

**Affiliations:** 1 Departments of Ophthalmology and Pharmacology, Emory University School of Medicine, Atlanta, GA; 2 Graduate Program in Biochemistry, Cell and Developmental Biology, Emory University School of Medicine, Atlanta, GA

## Abstract

**Purpose:**

Mice are commonly used in biomedical research, and euthanasia is an important part of mouse husbandry. Approved, humane methods of euthanasia are designed to minimize the potential for pain or discomfort, but may also influence the measurement of experimental variables.

**Methods:**

We compared the effects of two approved methods of mouse euthanasia on the levels of retinal dopamine. We examined the level of retinal dopamine, a commonly studied neuromodulator, following euthanasia by carbon dioxide (CO_2_)-induced asphyxiation or by cervical dislocation.

**Results:**

We found that the level of retinal dopamine in mice euthanized through CO_2_ overdose substantially differed from that in mice euthanized through cervical dislocation.

**Conclusions:**

The use of CO_2_ as a method of euthanasia could result in an experimental artifact that could compromise results when studying labile biologic processes.

## Introduction

Mice are commonly used in biomedical research today as genetic manipulation in mice is becoming more feasible and affordable. An important part of mouse husbandry is euthanasia, which should be performed as painlessly and efficiently as possible. There are several ways in which laboratory mice are euthanized, two of which include carbon dioxide (CO_2_)-induced asphyxia and cervical dislocation. CO_2_ overdose has been favored for euthanasia, because the lowering of pH in the cerebrospinal fluid (CSF) that occurs secondary to CO_2_ overdose is associated with anesthetic depth and insensibility to pain in humans [1]. Nevertheless, many laboratories regularly utilize cervical dislocation to euthanize laboratory mice. Although humane treatment of laboratory mice is important, the method of euthanasia may have a substantial effect on postmortem measurements of experimental variables and could significantly affect experimental results. Hence, in this study, we tested the hypothesis that CO_2_ overdose alters the retinal level of dopamine, an important regulator of visual function [2].

## Methods

### Animals

All animal experimental procedures were approved by Emory University’s Institutional Animal Care and Use Committee (IACUC) and conformed to the guidelines of the National Institutes of Health Guide for the Care and Use of Laboratory Animals. Five-month-old adult male 129/Sv mice (The Jackson Laboratory, Bar Harbor, ME) were used for this study. Animals were kept in a 12 h:12 h light-dark cycle. Lights were on from 7 a.m. to 7 p.m., and food and water were provided ad libitum. At approximately 1 p.m., all animals were euthanized by either CO_2_ overdose or cervical dislocation at the same time and in the same room. The room light intensity was about 700 lux. CO_2_ euthanasia consisted of gradual exposure to CO_2_ for 5 min as recommended by Pritchett et al. [3] for adult mice. Eyes were enucleated, and the retinas collected and stored immediately in −80 °C.

### Analysis of dopamine and its metabolite, 3,4-dihydroxyphenylacetic acid, with high-performance liquid chromatography

Levels of retinal dopamine and its major metabolite, 3,4-dihydroxyphenylacetic acid (DOPAC), were determined with ion-pair reverse-phase high-performance liquid chromatography (HPLC) with coulometric detection (guard cell at 0.6 V and coulometric analytical cell at 0.3 V) using a modification of the method as described by Pozdeyev et al. [4]. Two retinas from each mouse were homogenized in 200 µl of 0.2N HClO_4_ solution containing 0.01% of sodium meta-bisulfite and 25 ng/ml 3,4-dihydroxybenzylamine hydrobromide (internal standard). Samples were subsequently centrifuged at 15,000 ×*g* for 10 min at 4 °C, and 50 µl of the supernatant was used for HPLC analysis using an Ultrasphere ODS 5 µm 250×4.6 mm column (Beckman Coulter, Fullerton, CA) with a mobile phase containing 0.1 M sodium phosphate, 0.1 mM EDTA, 0.35 mM sodium octyl-sulfate, and 5.5% acetonitrile, pH 2.7. Standards of dopamine and DOPAC ranging from 2 to 20 ng/ml were analyzed with the samples. The precipitate obtained by centrifuging the homogenized retinas was resuspended in 100 µl of 1 N NaOH by sonication. An aliquot of 5 µl was used to estimate the amount of protein in each retina sample [5].

### Statistics

Comparisons of two groups were made with the Student *t* test using SigmaPlot 12 (Systat Software Inc., San Jose, CA). Error bars represent the standard error of the mean (SEM), and p<0.05 was considered significant.

## Results

**Figure 1:**
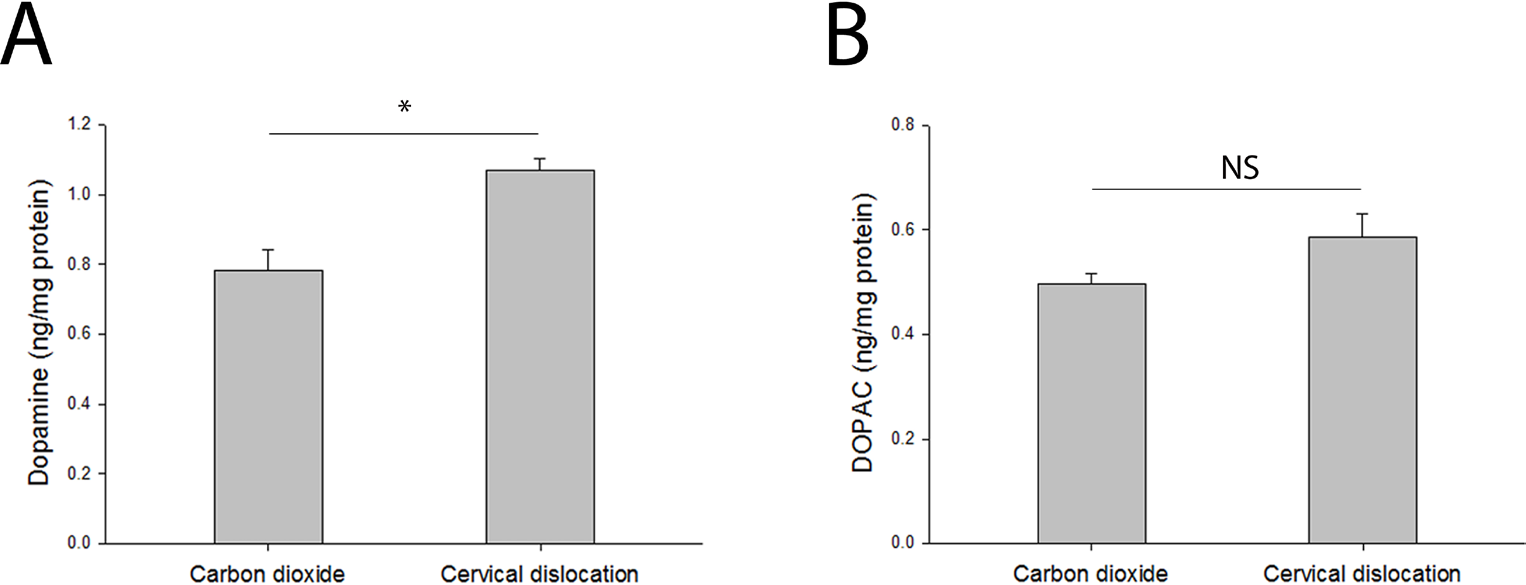
Retinal dopamine and 3,4-dihydroxyphenylacetic acid (DOPAC) levels in euthanized mice. **A**: Retinal dopamine levels in mice euthanized through carbon dioxide overdose were significantly different compared to those in mice euthanized through cervical dislocation (Student *t* test, p=0.003, n=5 for each group). **B**: Retinal 3,4-dihydroxyphenylacetic acid (DOPAC) levels between mice euthanized through carbon dioxide and those through cervical dislocation were similar (Student *t* test, p=0.119, n=5 for each group). The amount of retinal dopamine or DOPAC (ng) was normalized to the amount of retinal protein (mg) for each sample. Data expressed as mean± standard error of the mean (SEM).

The level of retinal dopamine was significantly different in mice euthanized with CO_2_ overdose compared to those in mice euthanized with cervical dislocation (Figure 1A; p=0.003). The normalized mean level of retinal dopamine of mice euthanized by the cervical dislocation method was 1.07 ng per mg of retinal protein, while the normalized mean level of retinal dopamine of mice euthanized by the CO_2_ overdose method was 0.78 ng per mg of retinal protein. However, the level of retinal DOPAC, dopamine’s major metabolite, was not statistically different between the two groups of mice (Figure 1B; p>0.05). The normalized mean level of retinal DOPAC of mice euthanized by the cervical dislocation method was 0.59 ng per mg of retinal protein, while the normalized mean level of retinal DOPAC of mice euthanized by the CO_2_ overdose method was 0.50 ng per mg of retinal protein.

## Discussion

The current study compared two methods of euthanasia approved by the American Veterinary Association on the level of retinal dopamine. We found that changing the euthanasia method from cervical dislocation to CO_2_ overdose was a major change in experimental condition that significantly altered the level of retinal dopamine.

Although it is not clear why dopamine levels are diminished in mice euthanized through CO_2_ overdose, we can conjecture that the mechanism involves factors of time, hypoxia, and respiratory acidosis that result from having excess CO_2_ in the blood. Dopamine turnover in the mouse retina exposed to light is rapid [6]. CO_2_-induced asphyxiation requires several minutes, and changes in oxygenation and pH could affect dopamine synthesis and metabolism during that period. In contrast, cervical dislocation is rapid, and retinas are typically dissected and frozen within 1–2 min. The rate-limiting step in dopamine biosynthesis is catalyzed by tyrosine hydroxylase, which requires molecular O_2_ for activity [7]. Thus, hypoxia associated with breathing concentrated CO_2_ could inhibit tyrosine hydroxylation and dopamine biosynthesis. Normally blood has a buffering capacity for CO_2_ in which water combines with CO_2_ to produce hydrogen and bicarbonate ions to keep the CO_2_ levels tightly regulated. However, when the capacity of the buffering system in the blood is exceeded, the excess CO_2_ leads to acidosis. Acidosis alters dopamine reuptake by affecting the activity of dopamine transporters [8,9]. As a result of hypoxia, extracellular dopamine could be favored to be metabolized by catechol-*O*-methyl transferase rather than monoamine oxidase, which requires oxygen to metabolize dopamine. These factors could explain why the level of retinal dopamine was affected while the level of retinal DOPAC was preserved.

In conclusion, we showed that mice euthanized with a CO_2_ overdose have significantly different retinal dopamine levels compared to mice euthanized with cervical dislocation. Thus, the use of CO_2_ as a method of euthanasia could result in an experimental artifact that could compromise results when studying labile biologic processes.
